# TLR9 is essential for HMGB1-mediated post-myocardial infarction tissue repair through affecting apoptosis, cardiac healing, and angiogenesis

**DOI:** 10.1038/s41419-019-1718-7

**Published:** 2019-06-17

**Authors:** Fang-Yuan Liu, Di Fan, Zheng Yang, Nan Tang, Zhen Guo, Shu-Qing Ma, Zhen-Guo Ma, Hai-Ming Wu, Wei Deng, Qi-Zhu Tang

**Affiliations:** 1 Department of Cardiology, Renmin Hospital of Wuhan University, 430060 Wuhan, PR China; 2 Cardiovascular Research Institute of Wuhan University, 430060 Wuhan, PR China; 3 Hubei Key Laboratory of Cardiology, 430060 Wuhan, PR China

**Keywords:** Apoptosis, Toll-like receptors, Angiogenesis

## Abstract

The poor prognosis of patients with acute myocardial infarction is partially attributed to a large number of cardiomyocyte apoptosis, necrosis, limited cardiac healing and angiogenesis, and cardiac dysfunction. Immune cells dysfunction leads to nonhealing or poor healing of wounds after acute myocardial infarction. Toll-like receptor 9 (TLR9) as an essential part of the innate immune system plays a vital role in regulating cardiomyocyte survival and wound healing. During hypoxia, High Mobility Group Box 1 (HMGB1), as the typical damage-associated molecular patterns (DAMPs) or alarmin, is rapidly released extracellularly and translocates from the nucleus to bind with cytoplasmic TLR9. However, the mechanism by which TLR9 interacts with HMGB1 and regulates myocardial damage remains unclear. Our current study found that the survival rate of TLR9KO mice with a higher rate of cardiac rupture was significantly lower than that in WT mice after 28 days post-operation. The effect of TLR9 knockout on insufficient wound healing in experimental MI was caused by a diminished number of myofibroblast and defective matrix synthetic capability. Moreover, the increased myocardial apoptotic cells and decreased angiogenic capacity were found in TLR9 knockout mice after MI. The results showed contrary in Recombinant Human High Mobility Group Box 1 (rhHMGB1) treated WT mice and similarity after applying rhHMGB1 in TLR9KO mice. This study demonstrates that TLR9 is essential for the repair of infarcted myocardium and interaction of HMGB1 and TLR9 is involved in the survival of myocardial cells, wound healing, and angiogenesis after myocardial infarction.

## Introduction

Myocardial infarction (MI) caused by coronary blockage may lead to cardiac arrhythmia, myocardial remodeling, and heart failure (HF), and remains the leading cause of cardiovascular-related deaths. Previous studies demonstrated that MI is usually accompanied by hypoxia, myocardial cell apoptosis, and death^1,2^. The duration of ischemia, the severity of the occlusion, the size or location of the infarction area, and the adaptation of cardiomyocyte to ischemic injury together determine the myocardial survival after MI^3^. However, along with permanent damage of the cardiomyocyte after myocardial ischemia, the local repairability of the heart is limited, and the role of immune cells and cardiac fibroblast is crucial for myocardial repair after injury.

The sterile immune system activated after acute myocardial ischemia relates to the initiation of signal transduction by pattern-recognition molecules (PRMs) including Toll-like receptors (TLRs), leading to the recruitment of neutrophils and mononuclear cells in infarcted area and ultimately the scar formation based on collagen deposition^4^. High Mobility Group Box 1 (HMGB1), a component of DAMPs and an endogenous ligand of TLR9, is an early warning signal of the innate and adaptive immune systems^5,6^. Recently studies have demonstrated the cardioprotection of exogenous HMGB1 on AMI through inhibiting myocardial inflammation, inducing angiogenesis, reducing infarct size, and improving myocardial function^7,8^. Premature intervention in inflammation and degradation of the cardiac matrix participate in the pathogenesis of cardiac rupture, as well as the delayed or defective formation of newly synthesized matrix proteins^9,10^. Differentiation of cardiac fibroblast, a repair process of proliferation and migration into the infarct area, reduces the risk of cardiac rupture after MI^10,11^. Angiogenesis as a crucial compensatory mechanism in the myocardial wound healing response of myocardial ischemia is also considered related to TLRs^11,12^.

TLR9 as an essential part of the innate immune system is expressed not only by multiple immune cells including monocytes, lymphocytes, etc., but by cardiomyocytes and cardiac fibroblast^13^. Although numerous studies have examined the effects of TLR9 ligands on MI^14,15^, the role of TLR9 deletion in ischemic cardiovascular disease has not been explored. Here, we performed an experimental MI on TLR9 knockout (TLR9KO) mice. We found that deficiency of TLR9 leads to insufficient fiber production, increased apoptotic cells, lack of angiogenesis, and leads to increased heart rupture and mortality. The above changes did not improve after myocardial injection of HMGB1. These data reveal a new role for TLR9 repair after MI and complement the protective mechanism of HMGB1 on MI.

## Results

### TLR9 levels were shown to be increased in mice and human hearts after MI and DCM

**Fig. 1:**
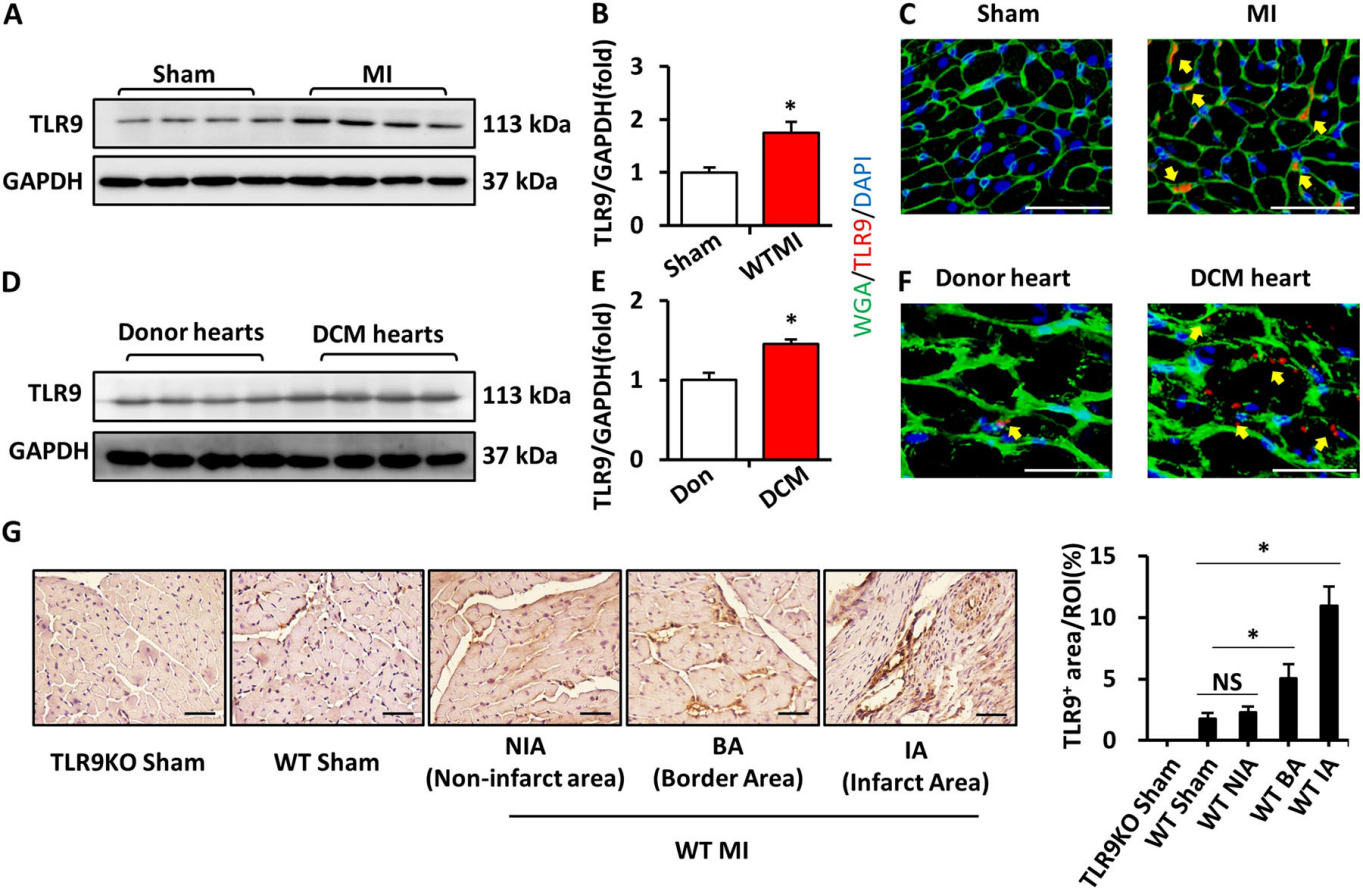
TLR9 expression was increased in mice post-MI hearts and human DCM hearts. **a**, **b** Protein expression level of TLR9 in the post-MI14d hearts as detected by western blot (*n* = 4). **c** Immunofluorescence and WGA staining analysis of TLR9 expression at the peri-infarct area in mice post-MI14d hearts (bar = 50 μm). **d**, **e** Protein expression level of TLR9 in the human hearts (*n* = 4). **f** Immunofluorescence and WGA staining analysis of TLR9 expression in human DCM hearts (bar = 50 μm). **g** Representative immunohistochemical analyses of TLR9 in sham-operated WT and TLR9KO mice and post-MI WT mice, including non-infarct area (NIA), border area (BA), and infarct area (IA) at day 14 post-MI (bar = 50 μm). TLR9 expression was quantified and compared (*n* = 6). TLR9 protein expression was normalized against GAPDH level (**a** and **d**). NS indicates not significant; *P < 0.05 vs. matched control

In order to detect the expression of TLR9 after MI, WT C57BL6/J mice were subjected to LAD surgery to establish a model of MI. The level of TLR9 expression in mice heart tissue at day 14 post-MI was increased (Fig. 1a, b). Immunostaining demonstrated the enhancing TLR9 expression at peri-infarct area in hearts post-MI and localization of TLR9 in heart post-MI was depicted in Fig. 1c. Similarly, we found increased TLR9 expression in the heart of DCM human hearts (Fig. 1d–f). Furthermore, immunohistochemical (IHC) analyses of WT mice heart confirmed that TLR9 was mainly expressed in the infarct and border areas at day 14 after MI with low expression in the Sham heart and non-infarct areas, and expression in TLR9KO sham mice could not be detected (Fig. 1g).

### TLR9KO aggravated MI-induced cardiac rupture, wall thinning, insufficient cardiac repair, and cardiac dysfunction

**Fig. 2:**
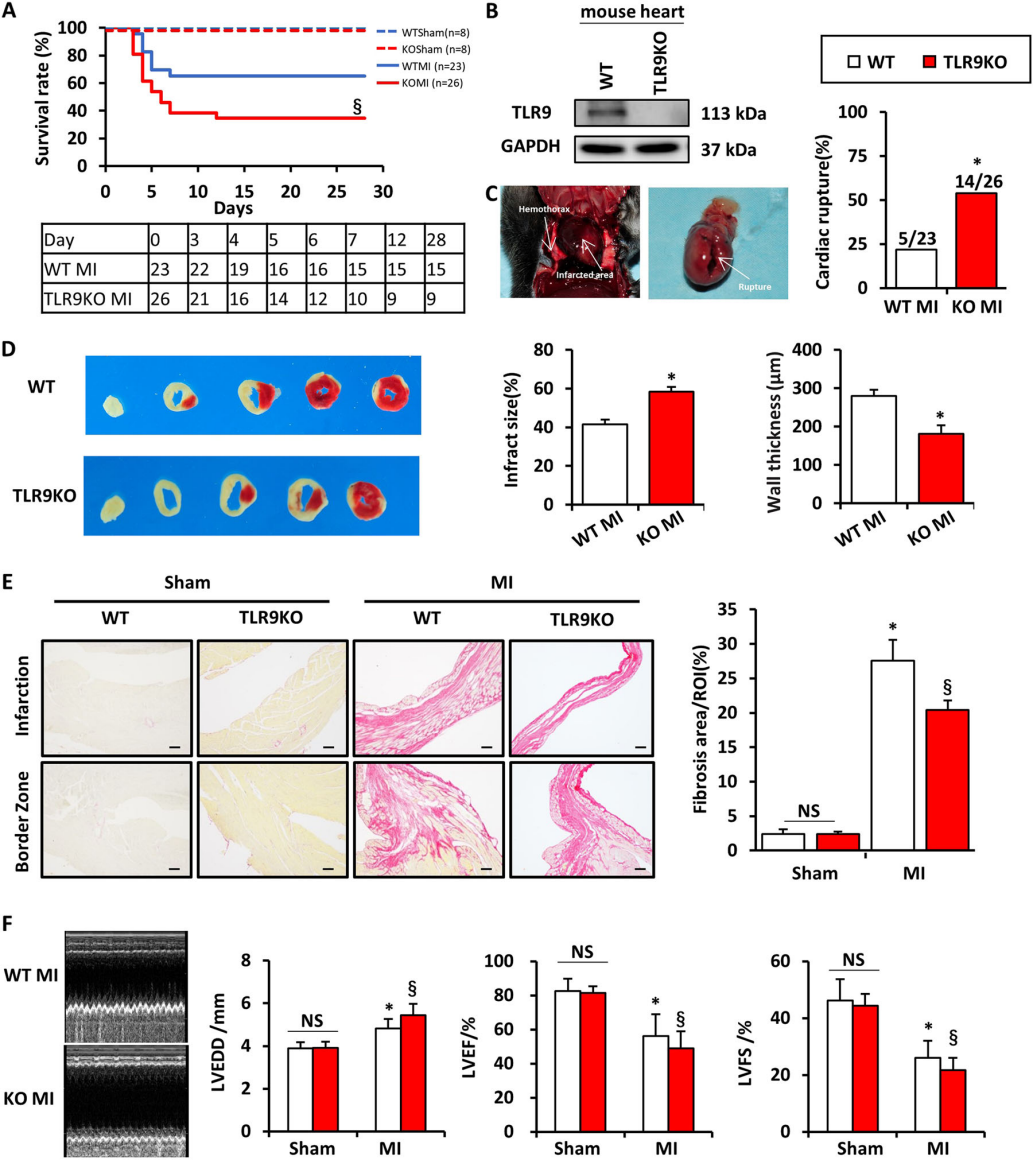
Effects of TLR9 deficiency on survival, ventricular rupture, myocardial fibrosis, and cardiac function. **a** Kaplan–Meier survival analysis post-MI in WT (*n* = 23) and TLR9KO (*n* = 26) mice and sham-operated in WT (*n* = 8) and TLR9KO (*n* = 8) mice analyzed by log rank test. **b** Protein expression level of TLR9 in the sham-operated WT and TLR9KO hearts as detected by western blot. **c** Representative images of cardiac rupture in TLR9KO mice post-MI and Incidence of cardiac rupture in WT and TLR9KO mice after LAD surgery. Data were analyzed by chi-squared test. Small slit in LV wall and evidence of hemothorax that was diagnosed as cardiac rupture in deceased mice. **d** TTC staining (left) in WT and TLR9KO mice at day 3 after MI and quantitative analysis of infarct size (middle) and wall thickness of the infarct regions (right) (*n* = 5–6). **e** Representative PSR staining sections in infarct and border areas of WT and TLR9KO mice heart at day 14 after MI (left; bar = 50 μm). Quantification of fibrotic areas at day 14 post-MI in WT (*n* = 6) and D2KO (*n* = 6) mice. **f** Evaluation of cardiac function in WT and TLR9KO mice. quantitative analysis of left ventricular end-diastolic diameter (LVEDD), fractional shortening (FS), ejection fraction (EF) at day 14 after the MI or sham operation (*n* = 6–12). NS indicates not significant; *P < 0.05 vs. WT sham group; ^§^*P* < 0.05 vs. WT MI group

WT and TLR9KO mice were subjected to the left anterior descending (LAD) artery ligation. Four weeks after operation, the survival rate in TLR9KO mice was lower than that in the WT mice post-MI (34.62% vs. 65.22%, respectively; log-rank test *P* = 0.0328, Fig. 2a), no sham-operated mice in both groups died. Western blot confirmed TLR9KO in TLR9KO mice (Fig. 2b). On autopsy for dead mice during the study, cardiac rupture was confirmed based on the blood coagulation in the chest cavity, a small slit in LV wall, or a wall perforation at a left ventricular infarct area. The wall perforation tended to occur more frequently in a central region of an infarcted left ventricle (LV) or a border zone between infarcted and non-infarcted myocardium, where wall strength became relatively weak. The incidence of cardiac rupture-induced death was significantly higher in TLR9KO mice than in WT mice (*P* < 0.05, Fig. 2c). Infarct size detected by 3%TTC staining showed significantly statistical difference in both group 3 days after MI (Fig. 2d). Wall thickness at the infarct area and fibrosis area detected by picrosirius red staining were significantly lower in TLR9KO mice than in the WT mice (Fig. 2d, e). Furthermore, echocardiographic parameters and cardiac function were analyzed in both group at day 14 after LAD surgery. Compared with WT mice, the acceleration of cardiac dilatation and deterioration of LV function were aggravated in TLR9KO mice (Fig. 2f).

### TLR9 deficiency was detrimental to wound healing and scar formation post-MI

**Fig. 3:**
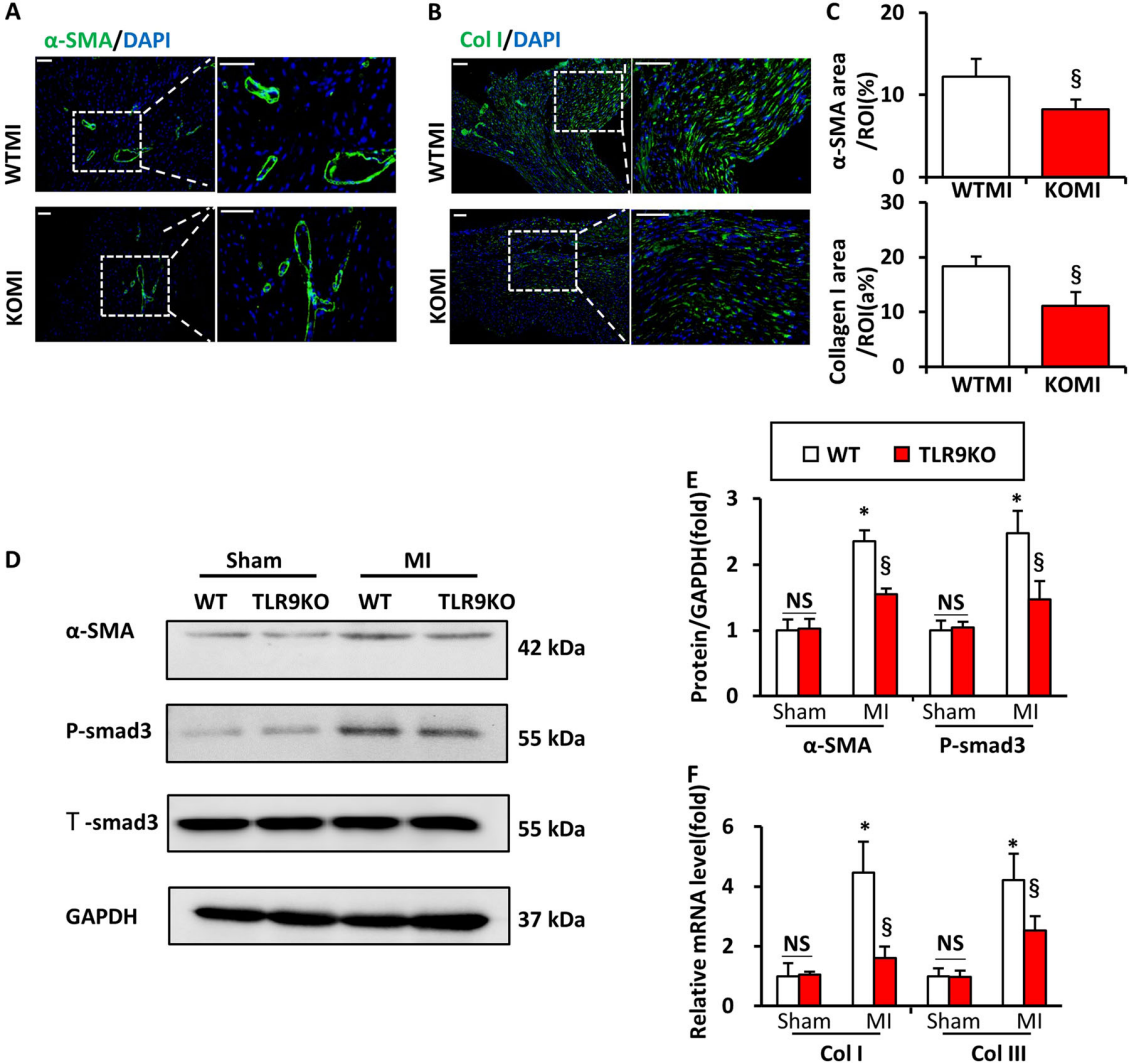
TLR9 deficiency was detrimental to scar formation and wound healing post-MI. **a**–**c** Immunofluorescence analyses of α-SMA (**a**, bar = 50 μm) and collagen I (**b**, bar = 50 μm) in the heart tissue at day 7 post-MI (*n* = 6). **d**, **e** α-SMA and smad3 protein expression at day 7 post-MI in the infarct area isolated from WT and TLR9 mice (*n* = 6). **f** Gene expression of collagen I (Col I) and collagen III (Col III) at day 14 after MI (*n* = 6). NS indicates not significant; *P < 0.05 vs. WT sham group; ^§^*P* < 0.05 vs. WT MI group

Next, to discover the mechanisms of the cardiac rupture, myocardial wound-healing and myofibroblast differentiation were investigated in the early stage after MI. The expression level of α-SMA and collagen I evaluated by immunofluorescencal analyses were significantly lower in TLR9KO mice than in the WT mice at day 7 post-MI (Fig. 3a–c). The protein levels of α-SMA and smad3 were significantly lower in TLR9KO mice than in WT mice at day 7 post-MI (Fig. 3d, e). Additionally, the mRNAs levels of collagen I and collagen III in the infarcted heart was determined to be lower in TLR9KO mice than in WT mice (Fig. 3f).

### TLR9 deficiency exacerbated early cardiomyocyte and fibroblast apoptosis after MI

**Fig. 4:**
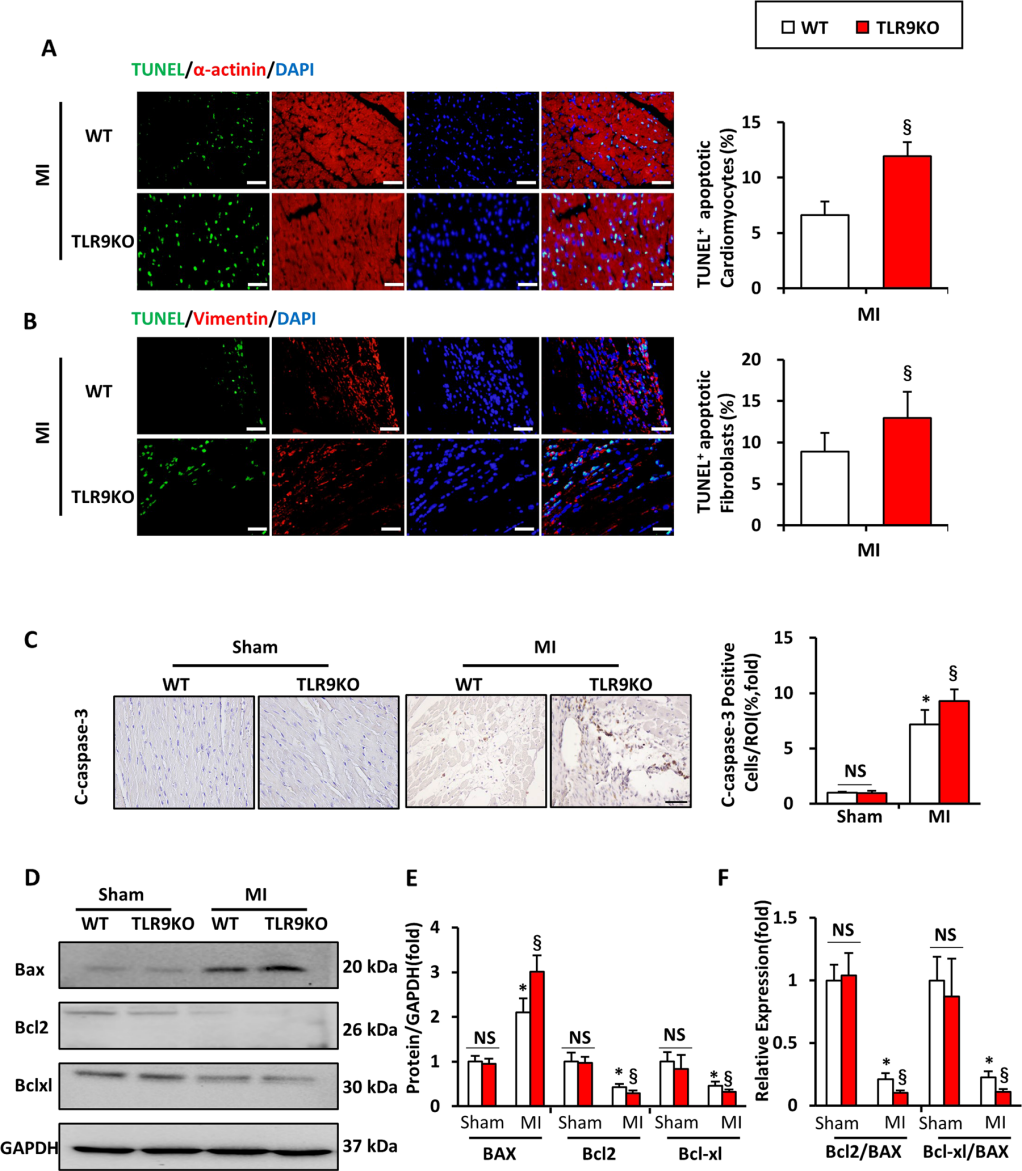
TLR9 deficiency aggravated cardiomyocyte and fibroblast apoptosis post-MI. **a** Representative TUNEL and α-actinin staining of cardiac myocytes in heart sections from WT and TLR9KO mice at day 3 after MI (*n* = 6; bar = 50 μm). Green color was TUNEL staining representing apoptotic cells; red color was the α-actinin staining representing cardiac myocytes; blue color was the cell nucleus stained by DAPI. **b** Representative TUNEL and vimentin staining in heart sections from WT and TLR9KO mice at day 3 after MI (*n* = 6; bar = 50 μm). Green color was TUNEL staining representing apoptotic cells; red color was the vimentin staining representing fibroblasts; blue color was the cell nucleus stained by DAPI. **c** Cleaved caspase-3 expression in the heart sections of the WT and TLR9KO mice post-MI after 7 days determined by immunohistochemistry (*n* = 3; bar = 50 μm). **d**–**f** Western blot was used to detect the protein levels of Bax, Bcl-2, and Bcl-xl in mice for each group, then the apoptotic index, Bcl-2/Bax, and Bcl-xl/Bax of each group were calculated and analyzed. NS indicates not significant; *P < 0.05 vs. WT sham group; ^§^*P* < 0.05 vs. WT MI group; ^&^*P* < 0.05 vs. WT MI mice in infarct area; ^#^*P* < 0.05 vs. WT MI mice in border area

Terminal deoxynucleotidyl transferase-mediated dUTPbiotin nick-end labeling (TUNEL) staining was performed to detect apoptosis at day 3 after MI in TLR9KO and WT mice (Fig. 4a, b). Immunostaining with anti-α-actinin and anti-vimentin antibody (to identify cardiomyocytes and fibroblasts, respectively) was also performed simultaneously (Fig. 4a, b). TUNEL staining revealed that the number of apoptotic cells was significantly higher in both cardiomyocytes and fibroblasts of the TLR9-KO mice than in the WT mice at day 3 post-MI. We further performed IHC staining of cleaved-caspase-3 in areas surrounding infarcted areas obtained from WT and TLR9KO mice at day 3 after MI. The positive cells of cleaved-caspase-3 detected by IHC staining in TLR9KO mice were significantly increased compared with WT mice at day 3 post-MI (Fig. 4c). In western blot, increased Bax and decreased Bcl-2 and Bcl-xl was detected in TLR9KO mice heart compared with that in the WT mice, which meant the higher ratio of Bcl-2/Bax and Bcl-xl/Bax in TLR9KO mice (Fig. 4d–f).

### Deficiency of TLR9 led to insufficient angiogenesis post-MI

**Fig. 5:**
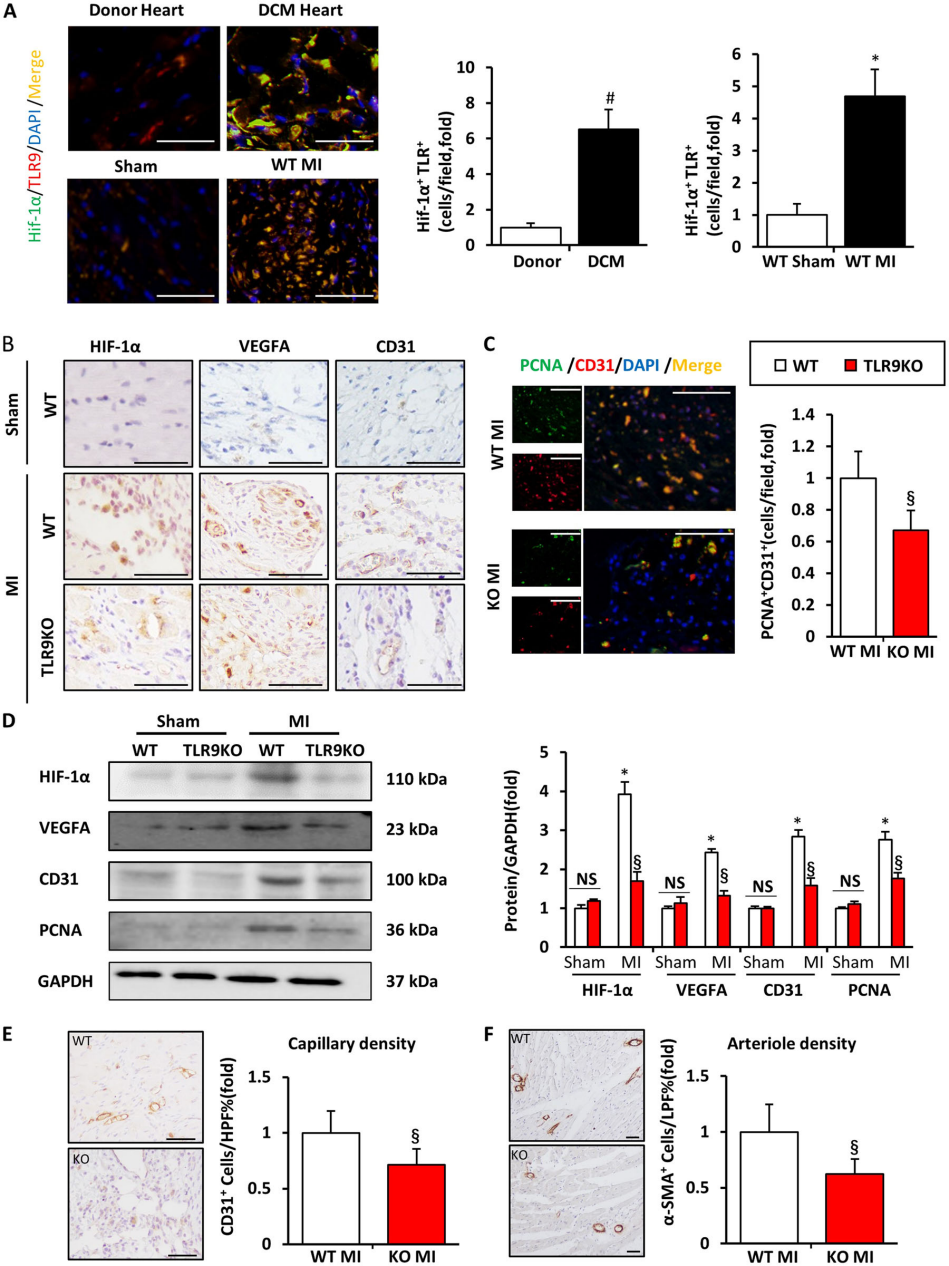
TLR9 deletion retarded angiogenesis post MI. **a** Representative immunostaining of TLR9 (red) and HIF-1α (green) in hearts, respectively, from human donor/DCM group (*n* = 4–6) and peri-infarct zones of mice hearts WT from sham/post-MI14d group (*n* = 5–6); The co-positive of HIF-1α and TLR9 cells were detected as yellow cells (bar = 50 μm). **b** HIF-1α, VEGFA, and CD31 expression in the heart sections of the WT and TLR9KO mice post-MI after 14 days determined by immunohistochemistry and quantitation of positive cells in injured hearts (*n* = 6). **c** Immunofluorescence analyses of CD31 (red) and proliferating cell nuclear antigen (PCNA, green) in peri-infarct zones of hearts from WT and TLR9KO mice at day 14 post MI; The co-positive of CD31 and PCNA cells were detected as yellow cells (*n* = 6, bar = 50 μm). **d** Representative western blots and quantification of HIF-1α, VEGFA, CD31, and PCNA in the cardiac tissues from the WT and TLR9KO mice post-MI after 14 days (*n* = 6). **e**, **f** Immunohistochemical staining with anti-CD31 and anti-α-SMA antibody in the border zone of the WT and TLR9KO mice heart at 14 days post MI, capillary densities (CD31-positive cells/HPF) and arteriole densities (α-SMA-positive cells/LPF) were acquired (*n* = 6, bar = 50 μm); *P < 0.05 vs. WT sham group; ^§^*P* < 0.05 vs. WT MI group; ^#^*P* < 0.05 vs. human DCM heart group

Several studies have shown that increased HIF-1α after hypoxia may be a critical stimulator of angiogenesis and cardiomyocyte survival^16–18^. To explore the role of TLR9 in angiogenesis after MI, we preformed double immunofluorescence staining for HIF-1α (green), TLR9 (red), and DAPI to stain nuclei (blue) in human and mice hearts. Both HIF-1α and TLR9 were extensively upregulated in the DCM human heart (*n* = 3) and WT mice heart in peri-infarct area (*n* = 6) at day 14 post-MI, as well as HIF-1α^+^/TLR9^+^cells (Fig. 5a). The number of positive cells of HIF-1α, VEGFA, and CD31 in TLR9KO mice was significantly lower than that in WT mice 14 days after MI (Fig. 5b). Then, endothelial proliferation of WT and TLR9KO mice hearts was determined by double immunostaining for proliferating cell nuclear antigen (PCNA) and CD31 (Fig. 5c). Similarly, compared with WT mice, endothelial proliferation was insufficient in TLR9KO mice. The protein of HIF-1α, VEGFA, CD31, and PCNA were lower in TLR9KO mice than that in WT mice (Fig. 5d). Furthermore, to determine capillary and arteriole densities, IHC staining for CD31 and α-SMA was examined in WT and TLR9KO mice heart sections taken from the border zone (Fig. 5e, f). Numbers of CD31-positive and α-SMA-positive cells were lower in TLR9KO than in WT mice (*P* < 0.05). These data suggest that neovascularization in the border zone at day 14 after LAD ligation was diminished after MI in TLR9KO mice.

### The protective effect of rhHMGB1 on MI was ineffective in TLR9-deficient mice

**Fig. 6:**
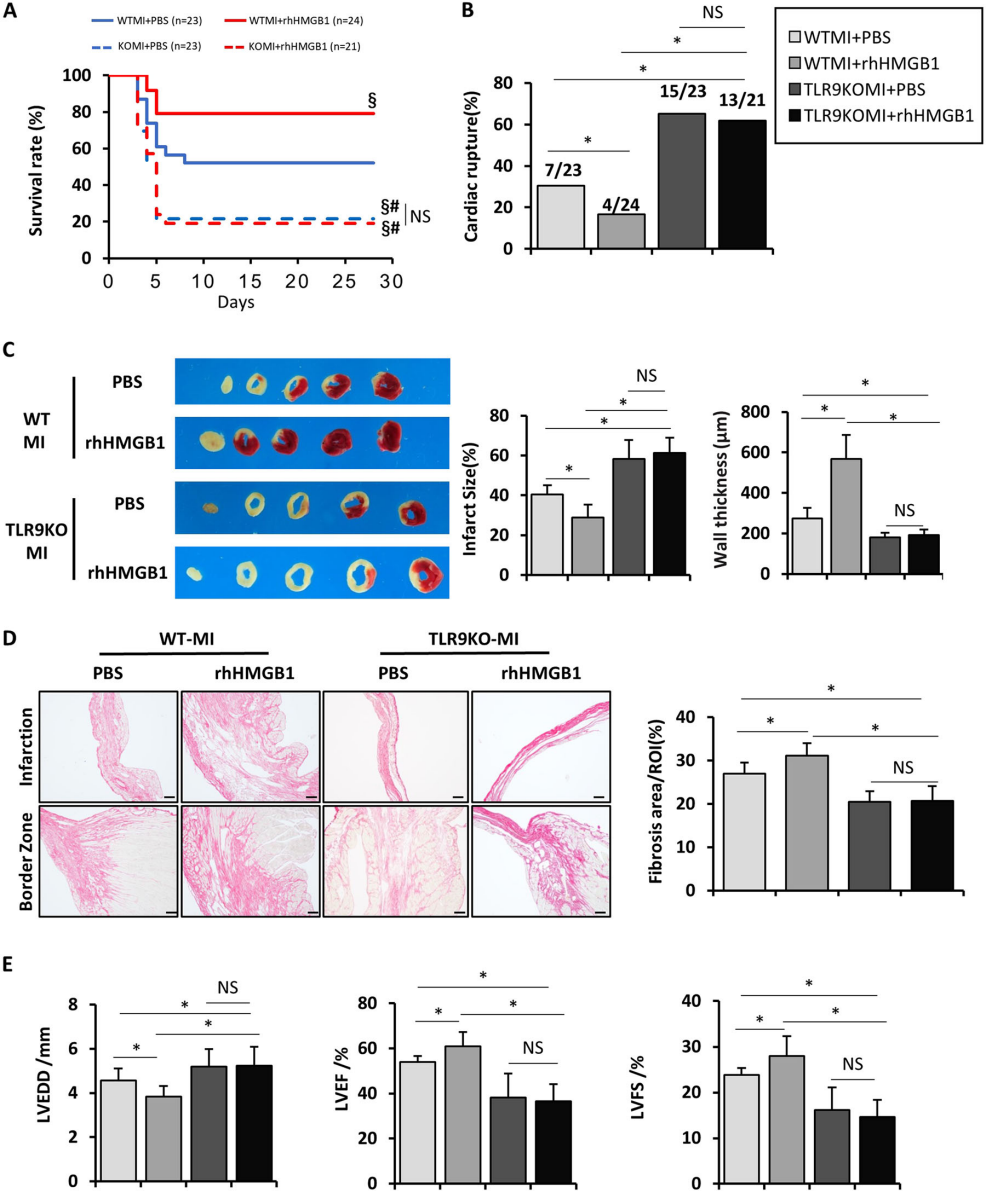
Effect of rhHMGB1 on WT and TLR9KO mice post-MI survival, ventricular rupture, myocardial fibrosis, and cardiac function. **a** Kaplan–Meier survival analysis post-MI in WT (*n* = 23), TLR9KO (*n* = 23), WT-rhHMGB1 (*n* = 24), and TLR9KO-rhHMGB1 (*n* = 21)mice. **b** Incidence of cardiac rupture in WT, TLR9KO, WT-rhHMGB1, and TLR9KO-rhHMGB1 mice at day 28 post-MI. Data were analyzed by chi-squared test. **c** TTC staining (left) in WT, TLR9KO, WT-rhHMGB1, and TLR9KO-rhHMGB1 mice at day 3 after MI and quantitative analysis of infarct size (middle) and wall thickness of the infarct regions (right) (*n* = 5–6). **d** Representative PSR staining sections of in WT, TLR9KO, WT-rhHMGB1, and TLR9KO-rhHMGB1 mice heart at day 14 after MI (left; bar = 50 μm). Quantification of fibrotic areas in infarct and border areas (middle, *n* = 6). at day 14 post-MI in in WT, TLR9KO, WT-rhHMGB1, and TLR9KO-rhHMGB1 mice (right, *n* = 6). **e** Evaluation of cardiac function in WT, TLR9KO, WT-rhHMGB1, and TLR9KO-rhHMGB1 mice. Quantitative analysis of left ventricular end-diastolic diameter (LVEDD), fractional shortening (FS), ejection fraction (EF) at day 28 after the MI or sham operation (*n* = 6). NS indicates not significant; *P < 0.05; ^§^*P* < 0.05 vs. PBS-treated WT MI group; ^#^*P* < 0.05 vs. rhHMGB1-treated WT MI group

As known protective effect of rhHMGB1 on MI and interaction between HMGB1 and TLR9^19–21^, intracardiac injection of rhHMGB1 was performed in the WT and TLR9-deficient mice before the LAD ligation. 28 days after operation, the survival rate in rhHMGB1-treated WT mice was significantly increased compared with that in other group mice (Fig. 6a): PBS-treated WT mice (79.17% vs. 52.17%, log-rank test *P* = 0.0447); PBS-treated TLR9KO mice (79.17% vs. 21.74%, log-rank test *P* < 0. 0001); rhHMGB1-treated TLR9KO mice (79.17% vs. 19.05%, log-rank test *P* < 0.0001). Surprisingly, there was no difference with PBS-treated TLR9KO and rhHMGB1-treated TLR9KO with in survival rate (21.74% vs. 19.05%, respectively; log-rank test *P* = 0.9689). The protection of rhHMGB1 in cardiac rupture-induced death was ineffective in TLR9KO mice (Fig. 6b). Wall thickness at the infarct area, and collagen volume fraction in the infarct and border areas were significantly higher in rhHMGB1-treated WT mice than that in another group, whereas Infarct size was decreased. However, there was no effect of rhHMGB1 on TLR9KO mice (Fig. 6c, d). The retardation of cardiac dilatation and improved cardiac function were analyzed in rhHMGB1-treated WT mice at day 14 after MI (Fig. 6e) compared with PBS-treated. Unfortunately, treatment with rhHMGB1 did not ameliorate cardiac function when compared with PBS-treated in TLR9KO mice.

### rhHMGB1 failed to enhance cardiac repair and reduce apoptosis in TLR9KO mice after MI

**Fig. 7:**
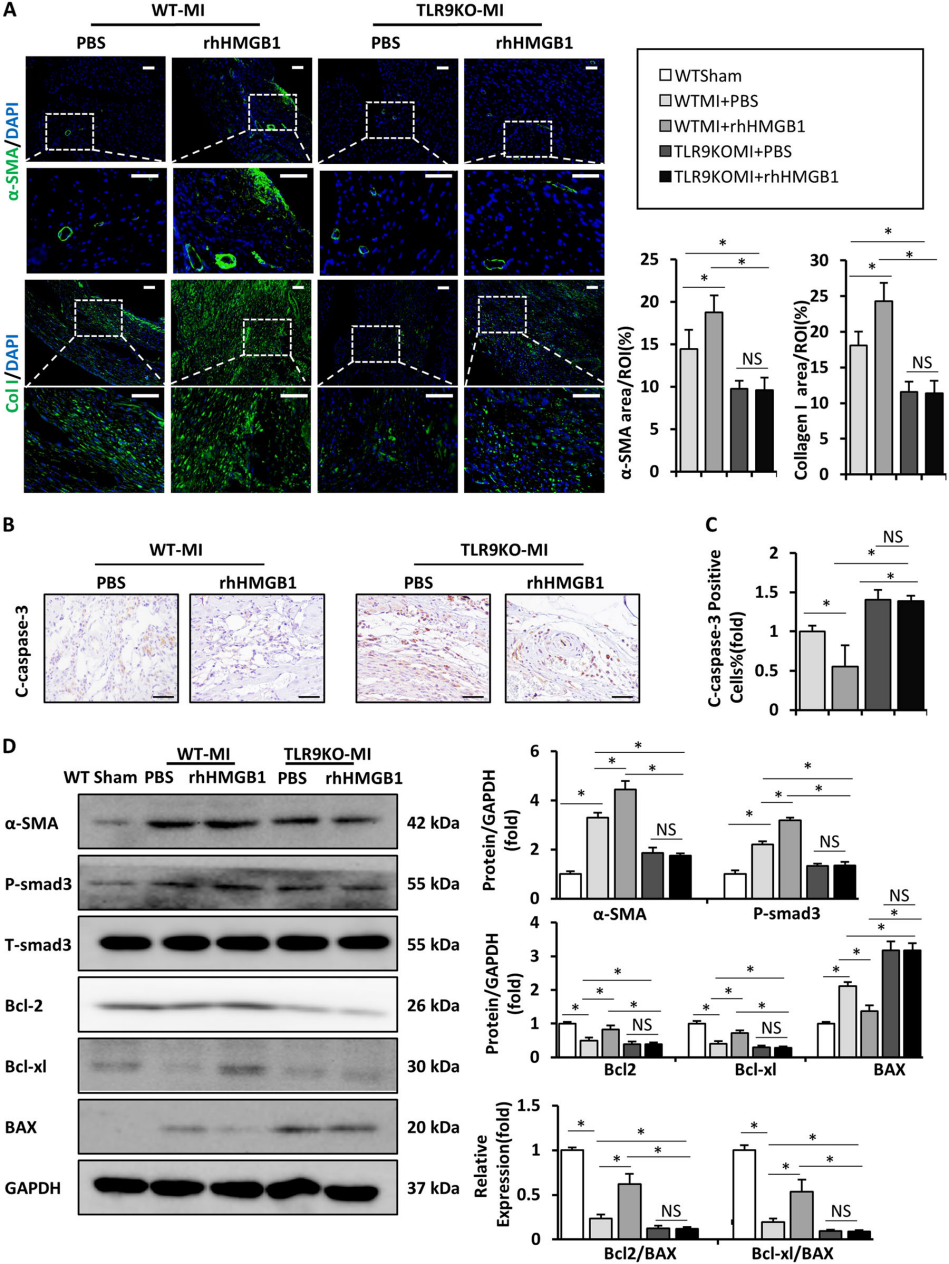
Effect of rhHMGB1 on WT and TLR9KO mice post-MI scar formation, wound healing, and cardiomyocyte and fibroblast apoptosis. **a** Immunofluorescence analyses of α-SMA (**a**, bar = 50 μm) and collagen I (**b**, bar = 50 μm) in the heart tissue at day 14 post-MI; **b**, **c** Cleaved caspase-3 expression in the heart sections of the WT and TLR9KO mice post-MI after 7 days determined by immunohistochemistry; **d** Western blot was used to detect the protein levels of α-SMA, smad3, Bax, Bcl-2, and Bcl-xl at day 14 post-MI in the infarct area for each group, then quantification and the apoptotic index, Bcl-2/Bax, and Bcl-xl/Bax of each group were calculated and analyzed. NS indicates not significant; *P < 0.05

To further investigate why the protective effect of HMGB1 ineffective in TLR9-deficient mice, We found that the expression levels of α-SMA and collagen I in immunofluorescence staining of rhHMGB1-treated TLR9KO mice heart did not increase as in rhHMGB1-treated WT mice at day 7 after MI, conversely, as low as PBS-treated KO mice (Fig. 7a). The protein levels of α-SMA and smad3 were significantly low in TLR9KO mice with or without rhHMGB1, which increased in rhHMGB1-treated WT mice in the meantime (Fig. 7d). IHC staining of cleaved-caspase-3 showed fewer positive cells in rhHMGB1-treated WT mice compared to others at day 3 after MI, and no significant difference between PBS or rhHMGB1-treated TLR9KO mice (Fig. 7b, c). As before, we detected the protein of Bax, Bcl-2, and Bcl-xl in all group heart (Fig. 7d). The lower expression of Bax and higher expression of Bcl-2, Bcl-2/ Bax, and Bcl-xl/ Bax ratio exhibited by rhHMGB1-treated WT mice did not appear in both TLR9KO mice (Fig. 7d).

### Angiogenesis in TLR9-deficient mice was not increased post-MI after intramyocardial injection of HMGB1

**Fig. 8:**
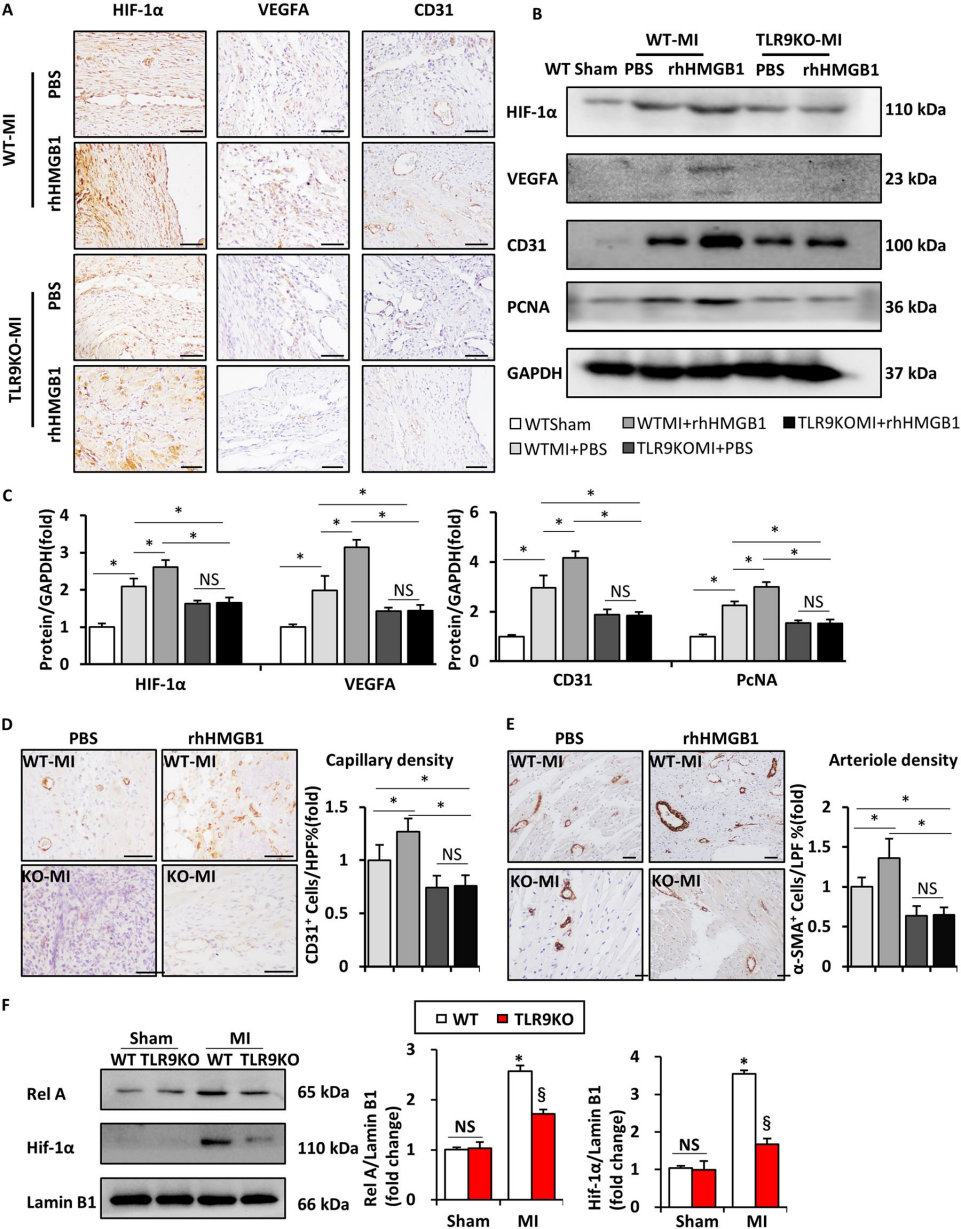
Effect of rhHMGB1 on WT and TLR9KO mice post-MI angiogenesis. **a** HIF-1α, VEGFA, and CD31 expression in the heart sections of the WT and TLR9KO mice post-MI after 14 days determined by immunohistochemistry (bar = 50 μm). **b**, **c** Representative western blots and quantification of HIF-1α, VEGFA, CD31, and PCNA in the cardiac tissues from the WT and TLR9KO mice post-MI after 14 days (*n* = 6). **d**, **e** Immunohistochemical staining with anti-CD31 and anti-α-SMA antibody in the border zone of the WT and TLR9KO mice heart at 14 days post-MI, capillary densities (CD31-positive cells/HPF) and arteriole densities (α-SMA-positive cells/LPF) were acquired (*n* = 6, bar = 50 μm). **f** Nuclear extracts were prepared at WT and TLR9KO mice hearts and analyzed by western blot for the nuclear accumulation of RelA and HIF-1a (*n* = 6). NS indicates not significant; *P < 0.05; ^§^*P* < 0.05 vs. WT MI group

Then, we detect the increased number of positive cells of HIF-1α in rhHMGHB1-treated WT mice at day 14 after MI, as well as VEGFA and CD31, which were all significantly decreased in TLR9KO mice with or without rhHMGB1 treatment (Fig. 8a). In western blot, the protein of HIF-1α, VEGFA, CD31, and PCNA were increased in rhHMGB1-treated WT mice, whereas lower level in both TLR9KO mice, compared with PBS-treated WT mice (Fig. 8b, c). Then, we examined the capillary density and arteriole at border area in all group mice heart at day 14 after MI (*n* = 4 per group). The rhHMGB1-treated WT mice showed more capillaries (*P* *<* 0.05), whereas the PBS-treated and rhHMGB1-treated TLR9KO mice had fewer capillaries (*P* < 0.05) than the PBS-treated WT mice. There was no difference in capillary content in PBS-treated and rhHMGB1-treated TLR9KO mice (Fig. 8d). The increased arteriole density in rhHMGB1-treated WT mice did not appear in rhHMGB1-treated TLR9KO mice (Fig. 8e). Based on co-immunofluorescent staining, we identified co-localization of TLR9 with HMGB1 in the human DCM and WT MI mice hearts at day 14 after MI (Supplement 1A). After IgG and TLR9 were IP, HMGB1 was not detected in the WT sham mice. However, HMGB1 was detected in the WT MI mice when TLR9 was IP(Supplement 1B). To further explore the molecular mechanisms underlying TLR9-regulated HIF1α, we examined the nuclear translocation of RelA and found that MI-induced nuclear translocation of RelA was inhibited in the hearts from TLR9KO mice (Fig. 8f).

### TLR9 activation showed the protection of heart in WT mice after MI

To extend functional analyses and translate our findings to a clinical application, we used a complementary approach. WT mice were treated systemically with TLR9 ligand, CpG-ODN 1826 (ODN-1826) before LAD ligation. The survival rate in ODN-1826-treated WT mice was higher than that in the PBS-treated WT mice post-MI (34.62% vs. 65.22%, respectively; log-rank test *P* = 0.0349, Supplement 2A). Infarct size detected by 3%TTC staining showed less in ODN-1826-treated mice 3 days after MI (Supplement 2B, C). Wall thickness at the infarct area and fibrosis area detected by picrosirius red staining were higher in ODN-1826-treated WT mice than in the PBS-treated WT mice at day 14 post-MI (Supplement 2D–F). The protein levels of α-SMA and smad3 were significantly higher in ODN-1826-treated WT mice than in PBS-treated WT mice at day 7 post-MI (Supplement 2G), which indicated the collagen-repair promotion of TLR9 ligand after MI. For apoptosis, decreased Bax and increased Bcl-2 and Bcl-xl was detected in ODN-1826-treated WT mice heart compared with that in the PBS-treated WT mice at day 3 post-MI, which meant the lower ratio of Bcl-2/Bax and Bcl-xl/Bax in ODN-1826-treated WT mice (Supplement 3A–C). For angiogenesis, the protein of HIF-1α, VEGFA, CD31, and PCNA were higher in ODN-1826-treated WT mice than that in PBS-treated WT mice at day 14 post-MI detected by western blot (Supplement 3D, E). Numbers of CD31-positive and α-SMA-positive cells were higher in ODN-1826-treated WT mice (Supplement 3F, G). These results demonstrate the promotion of TLR9 ligand on angiogenesis after MI.

## Discussion

Left ventricular free wall rupture (LVFWR) is a catastrophic complication and one of the most common causes of death following acute MI^22,23^. Therefore, a more comprehensive and thorough understanding of the intrinsic repair properties of heart after myocardial ischemia is required to refrain from the cardiac rupture or to seek the less invasive methods to repair the myocardial injury. MI induces the necrotic cardiomyocytes passive release of endogenous DAMPs (TLRs, etc.), then, alarmins such as HMGB1 activate the innate immune response and mediate inflammation^24^. Recently, it has been demonstrated activation of inflammation after myocardial injury can initiate the reparative process and promote a rapid healing response by activating expression of genes encoding inducible enzymes, cytokines, and growth factors^9,25,26^.

A large number of fibroblast migrate to the damaged area under the influence of damage factors and various biologically active factors, where they are transformed into myofibroblast, that are α-SMA positive and facilitate wound contraction and closure, and begin to synthesize multiple extracellular matrices (ECM) for repair after myocardial injury^10,27^. Here, we found that the healing capability of cardiomyocytes after MI seemed to be reduced with the deficiency of TLR9, and the possibility of defective wound healing increases significantly as follows. The deficiency of TLR9 led to a decreased survival rate and increased mortality due to acute cardiac rupture, which was expressed as fewer myofibroblast formation and reductions of collagen I in the peri-infarct area, moreover, the thinner wall thickness and less fibrotic area. Prior studies and our results have documented the cardiovascular-protection of TLR9 ligand ODN 1826 in experimental MI^14^, we illustrated that the positive role of TLR9 ligand in wound healing. Therefore, the early activation of smad3 by TLR9 leads to the reparative fibrotic response in vivo, which resulted in normal accumulation of myofibroblast in the infarct area and synthesis and maturation of the extracellular matrix.

Apoptosis in the ischemic area is associated with an irreversible loss of cardiac function, while the early increase (within 3 days post-MI) in apoptosis is correlated mainly with inflammation^1,28^. Apoptosis can be initiated by the death receptor pathway (extrinsic) and the mitochondrial pathway (intrinsic), and both lead to activation of caspase-3. In the mitochondrial pathway, the Bcl-2 family mediates the release of pro-apoptotic proteins from mitochondria into the cytoplasm, which consist of anti-apoptotic (Bcl-2, Bcl-xl, etc.) and pro-apoptotic (mainly Bax) members. In TLR9KO mice we found increased expression of Bax and caspase-3 activation, whereas decreased anti-apoptotic proteins, Bcl-2, Bcl-xl, as well as Bcl-2/Bax ratio and Bcl-xl/Bax ratio. However, pro-apoptotic and anti-apoptotic proteins in TLR9 ligand-treated mice showed the opposite results. As TUNEL staining showed, the deficiency of TLR9 markedly increased MI‐induced cardiomyocyte and fibroblast apoptosis. This may explain the reduced collagen density observed in the myocardial infarct area and aggravated systolic dysfunction.

One of another characteristic of proliferative phase after AMI is angiogenesis to restore tissue perfusion and to improve myocardial survival: endothelial cells form capillary to provide oxygen and nutrients for wound healing^25^. HIF-1α plays a crucial part in cell proliferation, differentiation, angiogenesis, and vascular remodeling after myocardial ischemic-anoxic injury by regulating the expression of its related genes through binding to the specific target gene hypoxia response element, and thus supports cell survival in hypoxic conditions^29–31^. Here, we confirmed that the expression of HIF-1α is upregulated after MI in WT mice but downregulated in TLR9KO mice. Moreover, we detected the increased colocalization of HIF-1α and TLR9 in cardiomyocyte post-MI as immunofluorescence staining showed, which indicated the potential relationship between HIF-1a and TLR9. In response to alterations in oxygen tension, HIF-1α regulates the expression of angiogenic genes including vascular endothelial growth factor (VEGF)^29^. VEGF recognized as one of the most important pro-angiogenic factors and an effective mitogen of endothelial cells not only promotes endothelial cell migration but also stimulates smooth muscle cell proliferation and migration^32,33^. Also, the presence of immune cells, like macrophages and neutrophils leads to the release of cytokines that are capable of stimulating fibroblast growth factor and VEGF expression and is sufficient to produce angiogenesis^34,35^. Interestingly, TLRs activation not only initiates innate and adaptive immune responses but also regulates angiogenesis after binding to exogenous and endogenous ligands^8,14,36^. Our study showed that hypoxia induced the expression of VEGFA, and overexpression of VEGFA in WT mice promoted angiogenesis as demonstrated by an increase in capillary and arteriole densities. In TLR9-deficiency mice, HIF-1α was downregulated after MI compared with WT mice, and angiogenesis was suppressed as follows, which showed by less expression of VEGFA, insufficient endothelial cells proliferation and the decrease in capillary and arteriole densities in TLR9KO mice post-MI. Opposite results are showed in TLR9 ligand-treated mice. Based on the above, TLR9 may participate in angiogenesis through the expression of HIF-1α and promote angiogenesis during the early phase of AMI.

HMGB1, an endogenous ligand of TLR9, is rapidly released extracellularly and translocates from the nucleus of the necrotic cell to bind with cytoplasmic TLR9^6,37^. We identified co-localization of TLR9 with HMGB1 in the human DCM and WT MI mice hearts and direct interaction of TLR9 with HMGB1in WT MI mice. Various studies have illustrated that the application of exogenous HMGB1 before the release of endogenous HMGB1 immediately after MI can improve harmful outcomes of structure and function after MI through cardiac repair^7^, angiogenesis^8,38^, prevention of apoptosis and induction of autophagy to promote myocardial cell survival^39^, and improvement of cardiac function^40^. Our study showed that left ventricular injection of rhHMGB1 before the LAD ligation promote cardiac function, repair, and angiogenesis and attenuate apoptosis in WT mice, however, not in rhHMGB1-treated TLR9KO mice. We detected elevated expression of HIF-1α in WT mice intracardiac-injected by rhHMGB1. In TLR9KO mice, the sign of increased activation of HIF-1α induced by rhHMGB1 no longer existed and was replaced by the diminution of capillary and arteriole densities and reduced endothelial proliferation. Furthermore, we examined the nuclear translocation of RelA and found that MI-induced nuclear translocation of RelA was inhibited in the hearts from TLR9KO mice. Jordi et al. have reported that in LPS-stimulated macrophages revealed that RelA is recruited to the Hif1α and upregulated HIF1α expression^39^. These results suggest that TLR9 regulate HIF1α expression via RelA. The effects of TLR9 on NF-κB-dependent transcriptional up-regulation demonstrated in our present study are consistent with previous reports that TLR9 can activate NF-κB. Consistent with our observations, deletion of NF-κB p50 markedly increased the extent of expansive remodeling and aggravated systolic dysfunction post-MI. In the infarct area, a lower collagen density was observed^41^. We demonstrated that HMGB1 activity on TLR9 and the followed promotion of nuclear translocation of RelA are responsible for TLR9-regulated HIF1α expression. We provided evidence that deficiency of TLR9 abrogates protection effects of HMGB1 after AMI. Therefore, HMGB1-dependent cardioprotection after MI may depend on TLR9.

In summary, we have provided the first evidence for a role of TLR9 in mediating effects of wound healing, apoptosis, and angiogenesis during acute myocardial ischemia. Furthermore, this study contributes to our understanding of the role of TLR9 and HMGB1 in acute MI. Therefore, the development of the application potential of TLR9 agonist is a deeply worthwhile research topic.

## Materials and methods

### Human left ventricular samples

The LV of dilated cardiomyopathy (DCM) patients undergoing heart transplantation and the LV of the documented healthy donors as control samples were collected as previously reported. Informed consent was signed by all donors and their families. All human heart samples are dedicated for research purposes only, approved by the Renmin Hospital of Wuhan University Review Board, according to the Helsinki Declaration.

### Experimental animals and treatments

All animal care and experimental procedures were approved by the Animal Care and Use Committee of Renmin Hospital of Wuhan University (approval no. 2016005) in accordance with the guidelines for the Care and Use of Laboratory Animals published by the United States National Institutes of Health (NIH). All experimental procedures and data analysis were blindly performed by researchers unaware of the treatment allocation. Adult Male TLR9KO mice (C57BL/6 background, The Jackson Laboratory; 10–12 weeks old; body weights of 23.5–27.5 g) and WT C57BL/6 mice (10–12 weeks old; body weights of 23.5–27.5 g) acquired from the Institute of Laboratory Animal Science, Chinese Academy of Medical Sciences (Beijing, China) were used in this study. After acclimatizing laboratory environment for 1–2 weeks, mice were subjected to either a proximally LAD ligation or a sham operation. After anesthetized with 3% pentobarbital (50 mg kg^−1^, Sigma; i.p.) and left hemithoracotomy, the mice were performed LAD artery ligation with a 7-0 silk suture at the fourth intercostal space. Meanwhile, Sham-operated animals were subjected the same procedure except for LAD ligation. All surgical procedures and subsequent analyses were performed blindly. The cardiac function of surviving mice was randomly measured at 4 weeks after MI. Mice were euthanized with excess pentobarbital sodium (200 mg kg^−1^; i.p.) and all hearts were collected for subsequent experiments.

### Experimental protocol

The TLR9 ligand, CpG-ODN, was used to investigate the role of TLR9 agonist in AMI. WT mice were injected intraperitoneally with low dose CpG-ODN 1826 (*n* = 25, 10 μg/25 g body weight, InvivoGen) 1 h prior to LAD ligation^42^. Mice receiving phosphate buffered saline (PBS) were used as controls. To investigate the role of HMGB1 in AMI, mice were treated with 100 μl of PBS, 2.5 μg of recombinant human HMGB1 (rhHMGB1, R&D systems), or without rhHMGB1 by intramyocardial injection 1 h before the LAD ligation^19^.

#### Survival analysis

Survival analysis was performed for the mice after MI and the sham-operated mice for the 4 weeks study period. We excluded the mice, related to surgical-death, that died during the peri-surgical period, which is the time from the beginning of the surgery to 6 h after surgery. All mice that died before the end of the study period were autopsied to determine the cause of death. Cardiac rupture was indicated by intrathoracic blood coagulation and a small incision in LV wall^43^.

### Infarct size of MI

Hearts were excised and cut perpendicular to the base–apex axis into five slices, the infarct size were determined by TTC staining. The hearts which were stained deep-red color were considered as viable myocardium, while pale white color as necrosis myocardium. Area measurements were calculated by Image Pro-Plus 6.0 software to evaluate total area of the infarct area for each mice.

### Echocardiography and haemodynamic evaluation

After the mice were anaesthetized using isoflurane (1.5–2%) as depicted early^44^, M-mode images of LV were recorded to obtain LV end systolic diameter (LVESD), LV end diastolic size (LVEDD), LV ejection fraction (LVEF), and LV fractional shortening (LVFS), obtained parameters from at least three beats and average. Hemodynamic parameters were measured by microtip transducer catheter (SPR-839, Millar Instruments, Houston, TX, USA) embedded into the LV. The results were analyzed by the Millar pressure-volume system (MPVS-400)^44^.

### Histological analysis and immunostaining

The anaesthetized mice were directly euthanized after echocardiography and haemodynamic measurements obtained. The heart tissues were arrested in remission with 10% KCl and then fixed in 10% formalin, embedded in paraffin, and transversely cut into 5 μm sections and then deparaffinized and rehydrated. Sections were stained with picrosirius red-stain (PSR) to confirm the morphological effects, such as collagen volume and wall thickness of the scars, calculated as published previously^44^.

For immunohistochemical analyses, the mouse hearts were dissected and fixed in 4% formaldehyde solution and embedded in paraffin. Sections were stained with rabbit anti-TLR9 (abcam, ab17236), rabbit anti-cleaved caspase-3 (CST, 9661), rabbit anti-α-smooth muscle actin (abcam, ab5694), rabbit anti-HIF-1a (abcam, ab2185), rabbit anti-VEGFA (abcam, ab52917), and rabbit anti-CD31 (abcam, ab28364). After rinsing, anti-rabbit/mouse EnVisionTM+/HRP reagent (37 °C, 30–60 min) was used to incubate it, then it was reacted with 3,3′-diaminobenzidine (room temperature, 5–10 min) and counterstained with haematoxylin, and analyzed using the Nikon H550L microscope (Tokyo, Japan).

For immunofluorescence staining, frozen sections of human heart tissue or the paraffin-embedded sections of mice heart tissue were sealed with 8% sheep serum after washing in PBS, and incubated at 4 °C overnight with mouse anti-α-SMA primary antibodies (abcam, ab7817), rabbit anti-col1 primary antibodies (abcam, ab34710), rabbit anti-HIF-1α primary antibodies (abcam, ab2185), rabbit anti-TLR9 (abcam, ab211012, ab17236; santa cruz,sc-25468), rabbit anti-HMGB1 primary antibodies (abcam, ab79823), rabbit anti-CD31 primary antibodies (abcam, ab28364), and rabbit anti-PCNA (santa cruz, sc-7907). After being washed in PBS, the Alexa-Fluor-coupled secondary antibodies (room temperature, 1 h) were employed to incubate corresponding sections, then counterstained with 4′,6-diamidino-2-phenylindole (DAPI; Invitrogen, S36939).

### Quantitative real-time PCR and western blotting

Total RNA was extracted from RNA from heart tissues employing TRIzol (Invitrogen, 15596-026) on ice according manufacturer’s protocol. cDNA was generated with the Transcriptor First Strand cDNA Synthesis Kit (04897030001, Roche Diagnostics, Basel, Switzerland), then SYBR Green (Roche, 04707516001) was employed to amplify the transcripts. All primer details are listed in Table 1, while GAPDH served as the endogenous reference gene. Total and nuclear protein were extracted using a Nuclear and Cytoplasmic Protein Extraction Kit (Beyotime Biotechnology, Beijing, China). After evaluating the protein concentration by a BCA protein assay kit (Thermo Scientific), the protein extracts were fractionated by SDS/PAGE (50 μg). The protein transferred PVDF membranes (Millipore, IPVH00010) were incubated with primary antibodies rabbit anti-TLR9 (abcam, ab211012, ab17236; santa cruz, sc-25468), rabbit anti-α-smooth muscle actin (abcam, ab5694), rabbit anti-smad3 (phospho S423+S425, abcam, ab52903), rabbit anti-smad3 (abcam, ab40854), rabbit anti-Bax (cst, 2772), rabbit anti-Bcl-2 (abcam, ab196495), rabbit anti-Bcl-xl (cst, 2764P), rabbit anti-HIF-1α primary antibodies (abcam, ab2185), rabbit anti-CD31 primary antibodies (abcam, ab28364), rabbit anti-HMGB1 primary antibodies (abcam, ab79823), rabbit anti-NF-kB p65 (abcam, ab196495), rabbit anti-Lamin B1 (abcam, ab16048) rabbit anti-rabbit anti-PCNA (santa cruz, sc-7907), and rabbit anti-GAPDH (cst, 2118) at 4 °C overnight followed by incubation with secondary antibodies for 1 h at room temperature. The membranes were subsequently treated with ECL reagents (170-5061, BioRad, Hercules, CA, USA) and then visualized using Bio-Rad ChemiDoc™ XRS+according to the manufacturer’s instructions to analyze blots by Image Lab and normalized to GAPDH.

**Table 1:** Gene-specific primers used in quantitative RT-PCR

Species	Genes		Sequences
Mouse	GAPDH	Forward	5′-ACTCCACTCACGGCAAATTC-3′
		Reverse	5′-TCTCCATGGTGGTGAAGACA-3′
Mouse	Collagen I	Forward	5′-CCCAACCCAGAGATCCCATT-3′
		Reverse	5′-GAAGCACAGGAGCAGGTGTAGA-3′
Mouse	Collagen III	Forward	5′-GATCAGGCCAGTGGAAATGT-3′
		Reverse	5′-GTGTGTTTCGTGCAACCATC-3′

### TUNEL staining

Apoptotic cells were measured using terminal deoxynucleotidyl transferase dUTP nick-end labeling (TUNEL) assay by the ApopTag Plus Fluorescein in Situ Apoptosis Detection Kit (Millipore, S7111) according to the manufacturer’s instructions. The TUNEL-positive cells were assessed by the fluorescence microscope (OLYMPUS DX51) and quantified at high magnification (200×). The apoptotic cells were calculated as percentage of TUNEL-positive cells from total cell nuclei. At least four to five fields of view in areas surrounding infarcted areas of each slide were arbitrarily observed. Immunostaining with anti-α-actinin and anti-vimentin antibody was also performed simultaneously. The cardiomyocytes were identified by immunofluorescence with α-actinin antibody staining. Vimentin is a marker of fibroblasts.

#### Coimmunoprecipitation

Coimmunoprecipitation was conducted based on the manufacturer’s instructions for Protein A/G Magnetic Beads (HY-K0202, MedChemExpress, USA)^45^. Briefly, after washing the magnetic beads with binding/wash buffer, the heart tissues lysates were subsequently incubated with anti-IgG antibody (ab172730, abcam) and anti-TLR9 antibody (sc-25468, santa cruz) on a rocking platform at 4 °C overnight. After collected and washed five times with lysis buffer, the immunoprecipitated proteins were boiled with 1 × SDS loading buffer, separated using SDS–PAGE and then electrophoretically transferred to polyvinylidene difluoride membranes (Millipore). The membranes were blocked with 5% non-fat dry milk in Tris-buffered saline containing 0.1% Tween-20 and were immunoreacted with the indicated primary and secondary antibodies conjugated to horseradish peroxidase.

### Statistical analysis

Statistical analysis was performed using SPSS Statistics 22.0 (IBM) and GraphPad Prism 8. 0 (GraphPad Prism Software Inc., San Diego, CA, USA). All measurement data had normal distributions (*P* > 0.05) according to the one sample K–S test. Survival curves were obtained by the Kaplan–Meier method and compared by log-rank test. Data are expressed as mean ± S.D. and evaluated by an unpaired Student’s *t*-test for two groups and one-way ANOVA analysis of variance for multiple groups followed by post hoc Tukey test. *P* < 0.05 was considered as statistically significant.

## Supplementary information

Supplement 1 Colocalization and Interaction between HMGB1 and TLR9 after MI in WT mice

Supplement 2 Effects of TLR9 ligand on survival, collagen repair after AMI

Supplement 3 TLR9 ligand reduced the myocardial apoptosis and improve angiogenesis after AMI
