# Supplementary figures and images for: TLR9 is essential for HMGB1-mediated post-myocardial infarction tissue repair through affecting apoptosis, cardiac healing, and angiogenesis
Source: Cell Death Dis. 2019 Jun 17;10(7):480. doi: 10.1038/s41419-019-1718-7 (PMC6579765; doi:10.1038/s41419-019-1718-7)

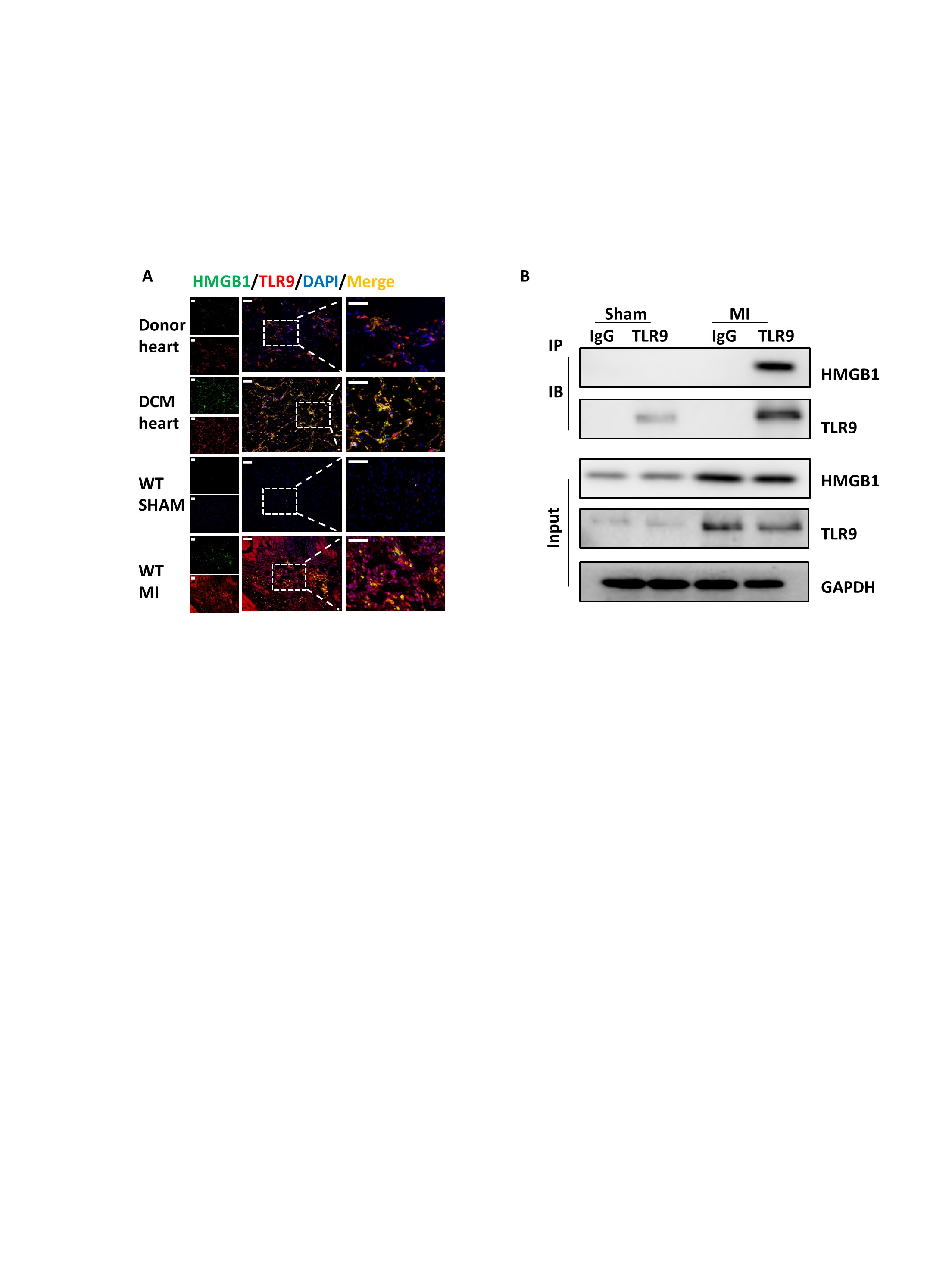

Supplement: Supplementary file 1 — Supplement 1 Colocalization and Interaction between HMGB1 and TLR9 after MI in WT mice [file 41419_2019_1718_MOESM1_ESM.jpg]

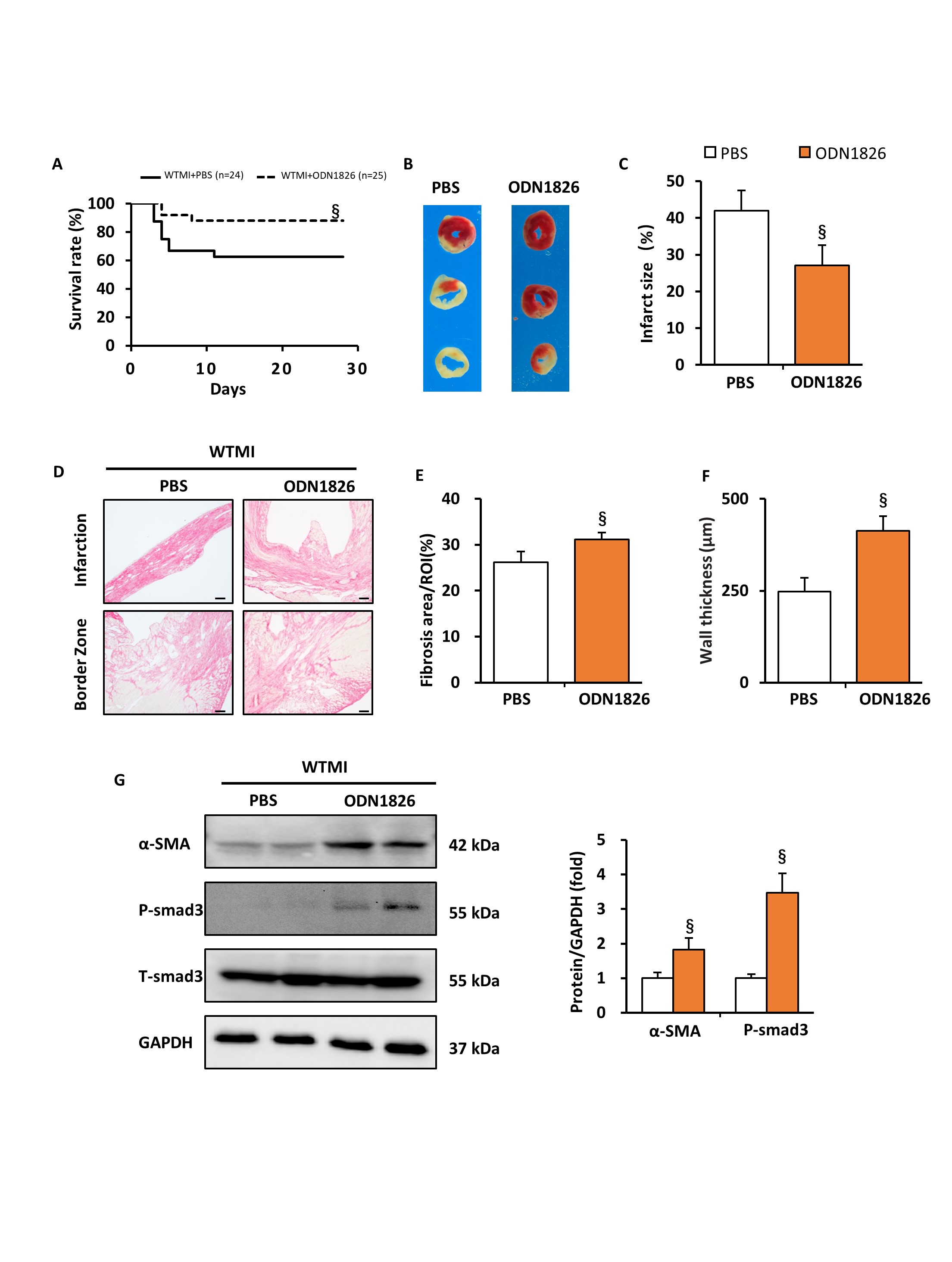

Supplement: Supplementary file 2 — Supplement 2 Effects of TLR9 ligand on survival, collagen repair after AMI [file 41419_2019_1718_MOESM2_ESM.jpg]

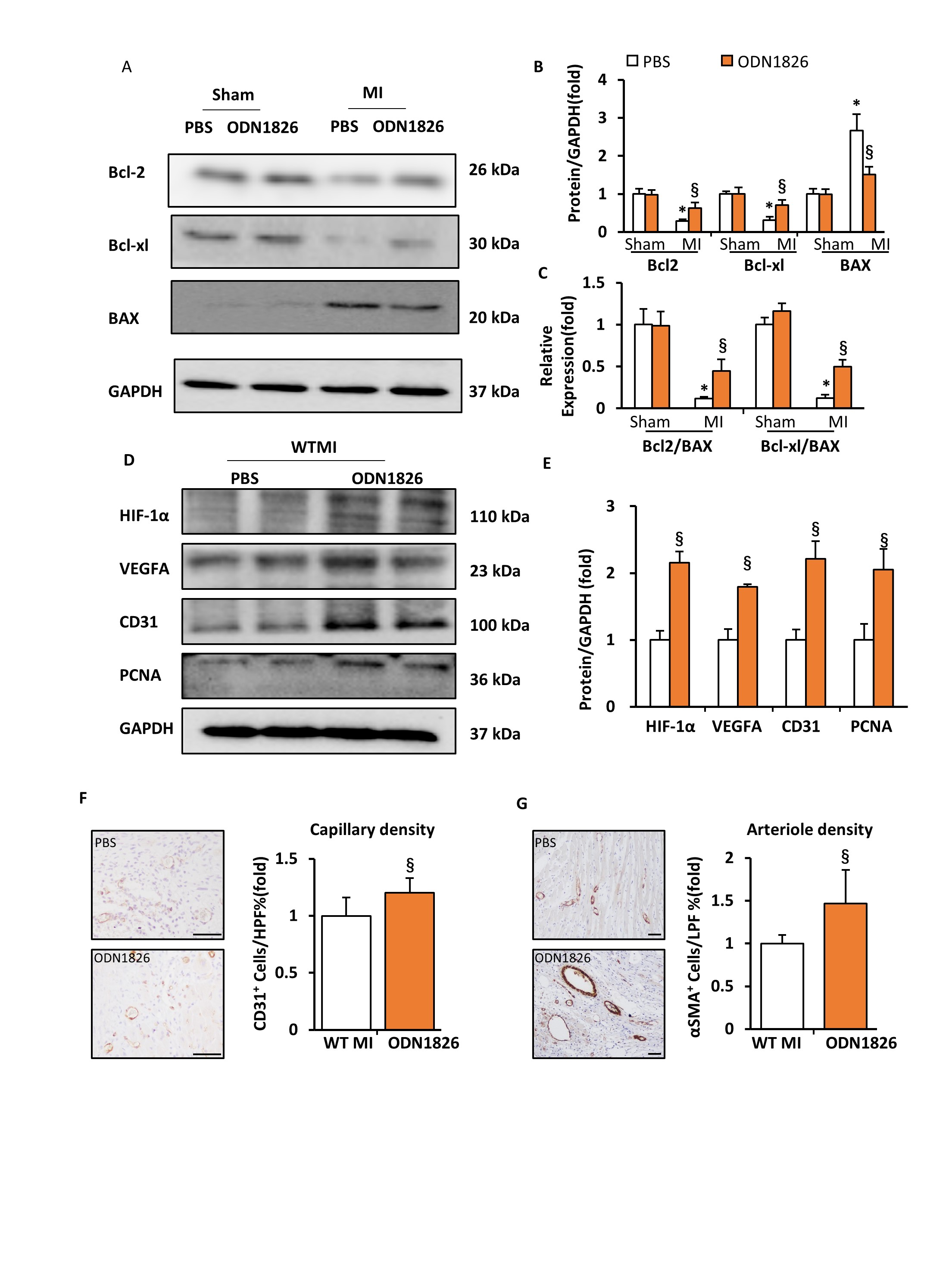

Supplement: Supplementary file 3 — Supplement 3 TLR9 ligand reduced the myocardial apoptosis and improve angiogenesis after AMI [file 41419_2019_1718_MOESM3_ESM.jpg]
